# The Impact of COVID-19 on African American Communities in the United States

**DOI:** 10.1089/heq.2020.0030

**Published:** 2020-10-30

**Authors:** Elena Cyrus, Rachel Clarke, Dexter Hadley, Zoran Bursac, Mary Jo Trepka, Jessy G. Dévieux, Ulas Bagci, Debra Furr-Holden, Makella Coudray, Yandra Mariano, Sandra Kiplagat, Ines Noel, Gira Ravelo, Michelle Paley, Eric F. Wagner

**Affiliations:** ^1^ College of Medicine, University of Central Florida, Orlando, Florida, USA.; ^2^ Community-Based Research Institute, Florida International University, Miami, Florida, USA.; ^3^ Department of Biostatistics, Robert Stempel College of Public Health and Social Work, Florida International University, Miami, Florida, USA.; ^4^ Department of Epidemiology and Robert Stempel College of Public Health and Social Work, Florida International University, Miami, Florida, USA.; ^5^ Department of Health Promotion and Disease Prevention, Robert Stempel College of Public Health and Social Work, Florida International University, Miami, Florida, USA.; ^6^ Center for Research in Department of Computer Vision (CRCV), Science, University of Central Florida, Orlando, Florida, USA.; ^7^ Division of Public Health, College of Medicine, Michigan State University, Flint, Michigan, USA.; ^8^ Center for Research on U.S. Latino HIV/AIDS and Drug Abuse, Robert Stempel College of Public Health and Social Work, Florida International University, Miami, Florida, USA.; ^9^ Affiliated Faculty, Department of Psychiatry and Behavioral Sciences, Miller School of Medicine, University of Miami, Miami, Florida, USA.

**Keywords:** COVID-19, African American, health disparities, infectious disease

## Abstract

**Purpose:** The purpose of this ecological study was to understand the impact of the density of African American (AA) communities on coronavirus disease 2019 (COVID-19) prevalence and death rate within the three most populous counties in each U.S. state and territory (*n*=152).

**Methods:** An ecological design was employed for the study. The top three most populous counties of each U.S. state and territory were included in analyses for a final sample size of *n*=152 counties. Confirmed COVID-19 cases and deaths that were accumulated between January 22, 2020 and April 12, 2020 in each of the three most populous counties in each U.S. state and territory were included. Linear regression was used to determine the association between AA density and COVID-19 prevalence (defined as the percentage of cases for the county population), and death rate (defined as number of deaths per 100,000 population). The models were adjusted for median age and poverty.

**Results:** There was a direct association between AA density and COVID-19 prevalence; COVID-19 prevalence increased 5% for every 1% increase in county AA density (*p*<0.01). There was also an association between county AA density and COVID-19 deaths; the death rate increased 2 per 100,000 for every percentage increase in county AA density (*p*=0.02).

**Conclusion:** These findings indicate that communities with a high AA density have been disproportionately burdened with COVID-19. To help develop effective interventions and programs that address this disparity, further study is needed to understand social determinants of health driving inequities for this community.

## Background

**FIG. 1.**
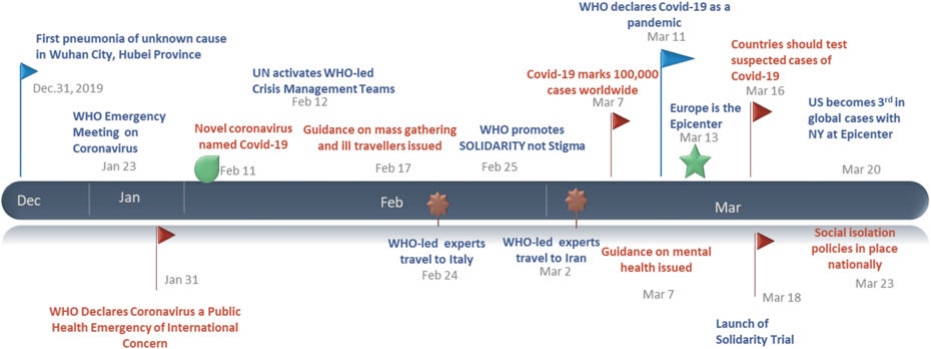
Timeline of COVID-19 pandemic events from December 2019 to March 2020. Flags and other images in the figure indicate an important point in the COVID-19 timeline. COVID-19, coronavirus disease 2019.

The coronavirus disease 2019 (COVID-19) was first reported in December of 2019 in Wuhan, China, and by January 31, 2020, the World Health Organization declared the outbreak to be a Public Health Emergency of International concern^1^ (see Fig. 1 for the timeline). By July 2020, in the United States there were >3 million COVID-19 cases and deaths surpassed 135,000.^2^

A notable key finding thus far of ongoing and evolving research is related to social disparities and health inequities in the United States that, compared with other races/ethnicities, blacks/African Americans (AAs) are disproportionately represented in the COVID-19 epidemic.^3,4^

This is not a unique experience for AA communities, as prevailing research provides evidence of blacks experiencing overall higher morbidities, earlier onset of morbidities, and higher mortality rates when compared with non-Hispanic whites in the United States.^5,6^ Specifically, AA adults (1) are 60% more likely to have diabetes than non-Hispanic white adults;^7^ (2) are responsible for 42% of new HIV cases even though they make up only 13% of the population^8^; (3) are 20% more likely to die from heart disease when compared with non-Hispanic whites^9^; (4) have a higher prevalence of asthma^10^; and (5) are 1.3 times more likely to be obese than non-Hispanic whites.^11^

Social determinants of health such as the places individuals live and work, their access to quality health care, and the resources to lead a healthy lifestyle play a major role in determining health status and health outcomes.^5,12^ In addition, a higher percentage of blacks live in densely populated communities and work as essential workers in service industries where they are at greater risk of exposure.^13,14^ Public health research shows that in comparison with their counterparts, blacks are more likely to be uninsured or underinsured and as a result have lower access to quality health care and tend to receive lower quality health care.^5,15^

Similar to influenza, for COVID-19, vulnerable and marginalized populations may be at greater risk for having more severe outcomes or death if they contract the virus. For COVID-19, vulnerable and at-risk populations (i.e., those with comorbidities such as asthma, diabetes, serious heart conditions, chronic kidney disease, and severe obesity) are at greater risk of negative outcomes.^16^ Owing to many social and economic factors, blacks are disproportionately affected by a number of these conditions^6,17^ and are more likely to have poor outcomes if COVID-19 is contracted. This study aims to understand the relationship between AA population density and other social determinants with COVID-19 prevalence and death rates nationally.

## Methods

An ecological analysis was completed using multivariable linear regression. The three most populous counties of each U.S. state and territory were identified and included in the data analysis, with the exception of Washington, DC, which is not considered a state so had only one entry, and California for which four counties were included (see Appendix A1 for the full list). This resulted in a final sample size of 152 counties and parishes. Data were analyzed for cases collected between January 22, 2020 and April 12, 2020. The data were sourced from USA Facts,^18^ and population estimates were derived from the U.S. Census.^19^

Independent variables were AA density (percentage of county/parish population who identified as AA), poverty level (percentage of county/parish population at the defined poverty level), and median age for the counties/parishes. Primary outcomes were the percentage of COVID-19 confirmed cases (prevalence) and rate of COVID-19 deaths per 100,000 persons/parish (death rate). Prevalence was used as an estimate of the existing burden of disease by county, and death rate provides inference on the health status of a community and the overall status of a public health system.^20^

Descriptive statistics, including overlapping frequencies, were used to describe COVID-19 prevalence, death rates, and AA density by county. Linear regression models were used to determine the relationship between AA population density and poverty, and, COVID-19 prevalence and death rates across U.S. counties and parishes. Both models for prevalence and death rate were adjusted for median age. All results were considered to be statistically significant at an alpha level of 0.05. Analyses were conducted using SPSS version 22.0.^21^ All data were from publicly available datasets therefore IRB approval was not necessary.

## Results

**FIG. 2.**
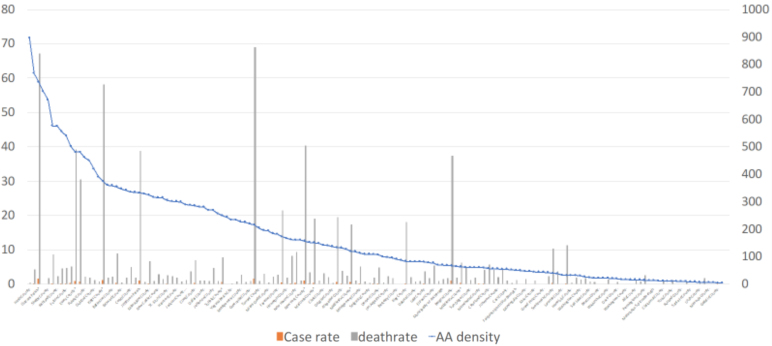
COVID-19 prevalence and death rates by AA density per county (*n*=152) in the United States, January–April 2020. Orange bars: percentage of COVID-19 confirmed cases in county population (case rate). Gray bars: COVID-19 deaths per 100,000 county population (death rate). Blue line: AA density in each county. AA, African American.

Two multivariable linear regression models were run to predict two health outcomes—confirmed COVID-19 cases or prevalence, and death rate. Illustration of the descriptive analysis (Fig. 2) demonstrates in some geographical areas or counties with higher AA density, there are peaks in prevalence and death rates.

### Prevalence

A multivariable linear regression was conducted to predict COVID-19 prevalence based on AA density and county/parish poverty level. There was a significant regression equation for this model: [*F*(3, 148)=5.7, *p*<0.01] with an R^2^ of 0.32. After adjusting for the county median age, there was a direct association between county AA density and COVID-19 prevalence; COVID-19 prevalence increased 5% for 1% increase in county AA density (*p*<0.01). There was no significant association between county poverty level and COVID-19 cases.

### Death rate

A multivariable linear regression was conducted to predict COVID-19 death rate based on AA density and county/parish poverty level. There was a significant regression equation for this model: [*F*(3, 148)=5.15, *p*=0.02] with an R^2^ of 0.30. After adjusting for the county median age, there was an association between county AA density and COVID-19 deaths; the death rate increased 2 per 100,000 for every percentage point increase in county AA density (*p*=0.02). Although not statistically significant, the data trended toward an association between county poverty level and the death rate (*p*=0.12). (See Table 1 for all results).

**Table 1.** Linear Regression for African American Density and Poverty Level on Coronavirus Disease 2019 Prevalence and Death Rate U.S. Counties/Parishes, January 22 to April 12, 2020 (*n*=152)

	Prevalence		Death rate	
	β	p	β	p
Median age	0.12	0.12	5.3	0.11
AA density	**0.05**	**<0.01**	**2.0**	**0.02**
Poverty level	0.08	0.23	4.2	0.12

Bold values indicate statistical significance.

AA, African American; COVID-19, coronavirus disease 2019.

## Discussion

According to this ecological study, in the United States, higher AA density had a stronger association with COVID-19 prevalence and death than higher median age in a community. These study findings support earlier research that suggested differences in cases, hospitalizations, and deaths could be due to the high prevalence of comorbidities among AAs.

Recently, since the relaxation of social distancing guidelines, spikes in cases have been observed in some cities, disrupting the flattening of the U.S. epidemiological COVID-19 curve.^22,23^ The possibility of a continued surge in the fall will be reliant on several factors, including relaxation of policies related to mitigation and social distancing, and possibly also an increase in travel-related/imported cases due to less travel restrictions.^24^

In the current surge, the most at-risk vulnerable populations, including people of color, continue to be disproportionately represented, more so than during the first outbreak of COVID-19. Stark disparities between how AA communities fare during the epidemic compared with other racial ethnic groups, can partially be attributed to social determinants of health (SDOH) that were not explored in this study but should be the focus of future research. Examples of SDOH relevant for AA individuals who potentially also live in counties with high AA density are high population density that can foster crowding—a known contributor to COVID-19 community spread; and insurance status that can impact an individual's access to and engagement in quality care.^5,15^ Consequently, parallel to existing models for barriers and facilitators of the HIV care continuum ranging from diagnosis to treatment,^25,26^ it is apparent that there are similar SDOH acting as barriers for AAs seeking COVID-19 care that need to be understood and addressed.^27^

For multiple reasons, AAs are more likely to be essential workers, placing them at a higher risk of exposure to the virus in their workplace.^28^ Furthermore, if diagnosed with COVID-19, AA essential workers may be forced into premature re-entry in the workforce before their illness resolves, thereby increasing the risk of those infected essential workers transmitting the virus to others.^3,4^ For younger healthier essential workers with low risk perception who can be asymptomatic or present with minimal symptoms, this becomes a dire problem that can increase the number of cases evolving as a result of wide community spread.^29^

In the foreseeable future, with the availability of vaccines and efficacious therapeutic lines,^30^ COVID-19 will likely become endemic, where it will constantly exist among minority populations,^31^ as is the other case with other historical pandemics.^32^ Part of working toward this “new normal” successfully and living with this novel coronavirus is to use all scientific and research efforts to minimize the number of fatalities. Implicit in this approach is ensuring that there is adequate testing in underserved areas such as AA communities where there may be more symptomatic cases.^33,34^ Screening and services should be comprehensively available regardless of insurance status or ability of the individuals to pay for medical care. Widespread availability of these services as well as vaccines and treatment when they are available will contribute to the reduction in overall incidence, transmission, and community spread.^34,35^

### Limitations

There are several limitations associated with this study. First, this is an ecological study, and there are several potential unmeasured confounding such as insurance status, employment risk, income, and education. Second, non-Hispanic whites were not included in the analysis as a possible reference group. A third limitation was that we did not account for testing differences between the counties. Finally, it is possible that prevalence estimates can be skewed depending on the availability of the test per county population.

## Conclusion

Study findings indicate an association between communities that have higher percentages of AAs and negative COVID-19 health outcomes, including higher prevalence and higher death rates. In addition, although not statistically significant in this study, the data suggested that the odds of surviving the epidemic may also be related to poverty levels suggesting that other at-risk minority populations, affected by poverty, may also be disproportionately affected.^36^ Further comprehensive analysis is needed to understand state, community, and individual levels of SDOH on COVID-19 health outcomes for all racial/ethnic minority and other vulnerable populations living in the United States.

## Abbreviations Used

AA: African American
COVID-19: coronavirus disease 2019
SDOH: social determinants of health

## Appendix A1

**List of counties included in analyses in alphabetical order (*n*=152)**

Ada County, ID

Allegheny County, PA

Allen County, IN

Arapahoe County, CO

Baltimore County, MD

Benton County, AR

Bergen County, NJ*

Berkeley County, WV

Bernalillo County, NM

Broward County, FL

Burleigh County, ND

Campbell County, WY

Canyon County, ID

Cass County, ND

Charleston County, SC

Chittenden County, VT

Clackamas County, OR

Clark County, NV

Cleveland County, OK

Cobb County, GA

Cook County, IL

Cumberland County, ME

Cuyahoga County, OH

Dakota County, MN

Dallas County, TX

Dane County, WI

Davidson County, TN

Davis County, UT

Denver County, CO

DeSoto County, MS

Doña Ana County, NM

Douglas County, NE

DuPage County, IL

East Baton Rouge Parish, LA

El Paso County, CO

Essex County, NJ*

Fairbanks North Star Borough, AK

Fairfax County, VA

Fairfield County, CT*

Fayette County, KY

Franklin County, OH

Fulton County, GA

Gallatin County, MT

Grand Forks County, ND

Greenville County, SC

Guilford County, NC

Gwinnett County, GA

Hamilton County, OH

Harris County, TX

Harrison County, MS

Hartford County, CT

Hawaii County, HI

Hennepin County, MN

Hillsborough County, NH

Hinds County, MS

Honolulu County, HI

Jackson County, MO

Jefferson County, AL

Jefferson County, KY

Jefferson Parish, LA

Johnson County, KS

Kanawha County, WV

Kent County, DE

Kent County, RI

Kenton County, KY

King County, WA

Kings County, NY*

Knox County, TN

Kootenai County, ID

Lake County, IL

Lake County, IN

Lancaster County, NE

Laramie County, WY

Lincoln County, SD

Linn County, IA

Los Angeles County, CA

Lyon County, NV

Macomb County, MI*

Madison County, AL

Maricopa County, AZ

Marion County, IN*

Matanuska-Susitna Borough, AK

Maui County, HI

Mecklenburg County, NC

Merrimack County, NH

Miami-Dade County, FL

Middlesex County, MA*

Middlesex County, NJ*

Milwaukee County, WI

Minnehaha County, SD

Missoula County, MT

Mobile County, AL

Monongalia County, WV

Montgomery County, MD

Montgomery County, PA

Multnomah County, OR

Municipality of Anchorage, AK

Natrona County, WY

New Castle County, DE

New Haven County, CT

New York County, NY*

Oakland County, MI*

Oklahoma County, OK

Orange County, CA

Orleans Parish, LA*

Palm Beach County, FL

Pennington County, SD

Penobscot County, ME

Philadelphia County, PA*

Pierce County, WA

Pima County, AZ

Pinal County, AZ

Polk County, IA

Prince George's County, MD

Prince William County, VA

Providence County, RI

Pulaski County, AR

Queens County, NY*

Ramsey County, MN

Richland County, SC

Riverside County, CA

Rockingham County, NH

Rutland County, VT

Salt Lake County, UT

San Diego County, CA

Santa Fe County, NM

Sarpy County, NE

Scott County, IA

Sedgwick County, KS

Shawnee County, KS

Shelby County, TN

Snohomish County, WA

St. Charles County, MO

St. Louis County, MO

Suffolk County, MA*

Sussex County, DE

Tarrant County, TX

Tulsa County, OK

Utah County, UT

Virginia Beach City, VA

Wake County, NC

Washington County, AR

Washington County, OR

Washington County, RI

Washington County, VT

Washington, DC

Washoe County, NV

Waukesha County, WI

Wayne County, MI*

Worcester County, MA

Yellowstone County, MT

York County, ME

*Counties with case rate peak and high AA density, except Bergen that has low AA density.
